# Telemedicine in Neurology: Challenges and Opportunities

**DOI:** 10.21203/rs.3.rs-3470381/v1

**Published:** 2023-10-27

**Authors:** Abrar Al-Faraj, Nene Ukonu, Omar Mohtar, Vibhav Jha, Dickson T. Chen, K. H. Vincent lau

**Affiliations:** Boston University Chobanian & Avedisian School of Medicine; Boston university School of Public Health; Boston University School of Medicine; Boston University; Boston University Chobanian & Avedisian School of Medicine; Boston University Chobanian &Avedisian School of Medicine

**Keywords:** Teleneurology, telemedicine, telehealth, barriers

## Abstract

**Objective:**

Our objective is to explore challenges encountered by neurologists with the use of telemedicine in neurology.

**Methods:**

A cross- sectional study via an anonymous survey to explore neurologists’ experiences with telemedicine. They survey was sent to randomly selected 200 participants from Academic Institutions in the United States. Descriptive statistics were reported as percentages for each survey question.

**Results:**

110 neurologists completed the survey. Fifty-one percent of neurologists stated that they experienced technological issues in (1%–20%) of telemedicine visits and 57% of neurologists needed technological assistance from informational technology support. With regards to the impact of limited neurological examination via telemedicine, 34% of neurologists agreed that the limited examination makes them worried that they are providing a suboptimal care to patients and 55% recommended a subsequent in-person visit (in 1%–20% of telemedicine visits) for further evaluation. Among the challenges that hindered patients’ ability to participate in telemedicine visits, 95% of neurologists rated patients’ technological challenges with setting up telemedicine to be the most common issue encountered, 37% of neurologists rated patient’s cognitive/mental disability to be the second most common challenge to complete telemedicine visits as well as availability of interpreter services for non-English speaking patients. Neurologists rated improving administrative support (39%), integration of EMR for video and telephone calls (37%), and sufficient time allotment to complete telemedicine visits (27%) to be the most important issues to address to optimize the use of telemedicine in neurology.

**Significance:**

Potential opportunities to improve neurologists’ experiences in telemedicine include improving technological support, integration of virtual platforms within the EMR, and adequate administrative support. Patients with cognitive/physical disabilities may need additional support to engage in the health system via telemedicine.

## Introduction

Telemedicine increases access to health care, especially to specialty care, to rural and underserved areas.^1, 2^ It also lowers patients’ physical and financial burden of traveling to appointments. ^2^ For physicians, it offers scheduling flexibility, and potentially improves work-life balance. In neurology, telemedicine has been shown to be non-inferior to in person evaluation.^3^ Therefore, it has been widely adopted by many neurological subspecialties such as stroke, epilepsy, neuro-oncology, and headache etc. ^3, 4,5^ Thus, telemedicine became an integral part of the health care system. However, with change comes many challenges. In neurology, research has been focused on examining the efficacy of telemedicine in comparison to in person evaluation, but research exploring challenges to telemedicine in neurology remains scarce. Neurology is unique because of the heavy reliance on physical examination, and the wide range of physical and cognitive abilities of patients (e.g., dementia, movement disorders or with neuromuscular disorders) which may affect the feasibility and efficacy of telemedicine in these subsets of patients. Identifying challenges in telemedicine in neurology will identify opportunities of improvement to facilitate the use of telemedicine in neurology. Therefore, our objective is to explore neurologists’ experiences with telemedicine in neurology.

## Materials & Methods

We conducted a cross-sectional study from March 23rd to August 5th, 2021. Currently, there is no validated tool that examines barriers and facilitators to telemedicine in neurology. Therefore, we developed a survey to explore neurologists’ experiences (facilitators and barriers) to telemedicine. The survey examined the following domains: (1) neurologists’ experiences in conducting telemedicine visits (e.g., technological difficulties, need for technological support to conduct telemedicine visit), (2) administrative staff support (e.g., faxing documents, preparation for virtual visits, medication prior authorization, and obtaining medical records), (3) patient’s cognitive disability, patient’s physical disability, use of interpreter services, and need for technology support from the family members, (4) impact of limited neurological examination on diagnosis and management, (5) areas of potential improvements in telemedicine to facilitate it’s use (e.g., time allotted for patient visits, administrative staff support, integration of electronic medical records with video/audio software).

The survey was distributed among three faculty at our institution (Boston University School of Medicine) for readability and clarity. The survey was revised and edited according to the feedback. Study data were collected and managed using REDCap electronic data capture tools hosted at Boston University and Boston Medical Center, CTSI 1UL1TR001430.11 This study was approved by the Institutional Review Board at Boston University School of Medicine.

### Study subjects:

The survey was randomly distributed to approximately 200 neurologists from academic neurology institutions in the United States. The institutions were selected randomly from the list of top 100 hospitals in the United States. Neurologists were recruited from only academic institutions for feasibility of recruitment. The first author disseminated the survey via email to the chairperson who disseminated the survey among their faculty. Three reminder emails were sent to the chairperson. Because of the exploratory nature of the survey, a power calculation was not performed.

## Statistical Methods:

Descriptive statistics were reported as frequencies and percentages for each survey question. SAS 9.4 was used to perform all statistical analyses.

## Results

A total of 159 neurologists participated in the survey, but only 110 completed the survey. Most participants were attending neurologists (85%), followed by fellows (11%), residents (2%), advanced practice providers (1%), and others (1%). The most common subspecialties participating in the study were vascular neurology (22%), epilepsy (19%), headache medicine (15%) and movement disorders (15%). Most participants indicated that they use telemedicine in the majority of their visits since the start of the COVID-19 Pandemic. other details of participants’ characteristics are in Table 1.

### Technological Challenges in Telemedicine:

Nearly half (51%) of neurologists stated that they experienced technological issues (e.g., interruptions by poor connection, inability to connect to video, inability to connect to audio, dropped calls, etc.) in 1%–20% of telemedicine visits. Thirty-one percent experienced technological issues in 21%–40%, 12% experienced technological issues in 41%–60% of their telemedicine visits, and only 6% experienced technological issues in 61%–80% of telemedicine visits. Furthermore, 57% of neurologists required assistance from informational technology (IT) support in 1%–20% of telemedicine visits, 14% required IT support in 21%–40% of telemedicine visits, and 3% required IT support in both 41%–60% and in 61%–80% of telemedicine visits.

### Patient-related barriers to telemedicine:

Neurologists were queried about the most common patient-related barriers to telemedicine (e.g., mental cognitive impairment, physical disability, technological support, and interpreter services for non-English speaking patients). Ninety five percent of neurologists rated patient’s difficulties in setting up telemedicine visit (technological support) as the most common difficulty encountered. Among other patient-related difficulties, 37% rated patient’s cognitive disability, 37% reported interpreter services limitations, and 18% reported patients’ physical disability as a limitation to telemedicine. A smaller percentage, 7%, rated other issues such as limited internet bandwidth, lack of patient cooperation with exams, patients not taking visits seriously, instability of the telemedicine platform, patients forgetting their appointments and inappropriate telemedicine setup. Details of patient-related barriers to telemedicine are in Table 2.

### Limited Neurological Examination:

We asked neurologists about the effect of limited ability to perform a neurological examination using the statement: “the inability to examine my patients makes me worried that I am providing a suboptimal care to my patients”. The responses were graded on a Likert scale (agree, strongly agree, neutral, disagree, strongly disagree). Thirty-four percent of neurologists agreed that the limited examination makes them worried that they are providing a suboptimal care to patients, 26% disagreed, 9% strongly agreed, 8% strongly disagreed and 24% were neutral to the statement. Furthermore, we asked neurologists about the percentage of their telemedicine visits in which they advised a subsequent in-person evaluation. Fifty percent of neurologists advised an in-person clinic evaluation in 1%–20% of their telemedicine visits, 16% advised it in 21%–40% of their telemedicine visits, and 11% recommended it in 41%–60% of their telemedicine visits. Smaller percentages, 8% and 11% of neurologists, recommended a subsequent in person evaluation in 61%–80% and 81%–100% of their telemedicine visits, respectively.

Neurologists were asked ‘how many additional studies do you order on average due to lack of In- person physical examination’. The majority (72%) reported no change in the studies ordered. Nineteen percent stated they order somewhat more additional studies due to a lack of in person physical examination, and 9% reported ordering somewhat fewer additional studies. Furthermore, 45% of neurologists reported somewhat more additional telemedicine- related responsibilities (e.g., extra documentation, additional paperwork, obtaining medical records, etc.), 13% reported significantly more responsibilities and 35% reported no change in administrative responsibilities. Smaller percentages reported significantly fewer responsibilities (2%) and somewhat fewer responsibilities (5%).

### Issues to Address to Improve Telemedicine in Neurology

Lastly, neurologists were asked to rank the most common issues to be addressed to improve their experiences in conducting telemedicine visits. The most frequently rated issues were increased administrative support (39%), integration of electronic medical records (EMR) for video and telephone calls (37%), and sufficient time allocation to complete telemedicine visits (27%). Other responses included: providing telemedicine devices to patients, improving internet access in rural areas, determining patient technological capability to conduct telemedicine, adding allotted time for patients requiring interpreter services, ensuring equal reimbursement for telemedicine and in person visits, virtual rooming for patients via medical assistants and other staff members, and improved patient preparation for the visit.

## Discussion

Our study provides important insights into the challenges encountered in telemedicine in neurology. Those challenges can be categorized into telemedicine infrastructure-related challenges and patient-related barriers.

### Telemedicine Infrastructure Barriers:

#### 1.1 Technology support

Nearly half of neurologists reported encountering technological difficulties that require IT support in 1%–20% of telemedicine visits. Our findings are consistent with existing literature that describes similar challenges related to technology support. (1) Common technological difficulties include virtual platform functionality, connection reliability, accessibility, and availability of technological support. (2), (3), (4), (5),(6) Suboptimal technology support can lead to many technical inefficiencies that may increase the workload on neurologists, as well as frustration and delay in the delivery of care.(2),(7) Establishing an effective telemedicine practice requires the development of strong technological infrastructure and use of adequate equipment (e.g., hardware, software, and trained personnel) that meet the requirement of the Health Insurance Portability and Accountability Act (HIPAA).(2), (7) It also requires effective technological implementation, training, and maintenance.(2) Furthermore, supporting telemedicine programs via IT requires a special set of skills compared to standard IT support. It requires trained personnel who have an understanding of how video conferencing works, networking, firewall, and basic personal computer skills, and can interface and communicate effectively with the clinical staff.(4)

#### 1.2 Limitation of Neurological examination:

Nearly one-third of neurologists agreed that the inability to perform an in-person neurological examination is a concern that they may be providing suboptimal care to their patients’. Nearly half of neurologists generally followed telemedicine visit with a subsequent in-person evaluation in 1%–20% of their telemedicine visits. Nonetheless, participants did not report significant changes in the percentage of ordering additional ancillary testing (e.g., MRIs) due to the limited neurological examination. Concerns about the impact of limited physical examination were reported in neurology as well as other subspecialties that have a heavy reliance on physical examination, such as neurosurgery (specifically spine surgery) and orthopedics.(2),(5),(4),(6) Notably, research showed that neurological examination via telemedicine is generally non-inferior to face-to-face evaluation. (8), (9),(10) However, our results showed that one third of neurologists have a negative perception about the impact of the limitation of neurological examination via telemedicine. And it is influencing their decision to have a subsequent in person visit. There have been creative efforts to overcome some of the barriers to performing physical examination via telemedicine, for example, Telemedicine Examination Medical Devices peripherals. (11) These medical devices (e.g., tele-ophthalmo-scope, video-otoscope) allow the transfer of data stream through a shared digital interface for review by the physician.(11) In neurology, some disease- specific examination tools may be helpful, for example, the Unified Parkinson’s disease rating scale (UPDRS).(12) A similar score can be developed to other neurological diseases. However, reliability and validation need to be established prior to the adoption of these tools.(12)

### Patient-related barriers to telemedicine:

Patient-related barriers to telemedicine are common.(13) The majority of neurologists reported that patients’ difficulty in connecting to telemedicine were the most common difficulty encountered during telemedicine visits. Similar findings were also reported in a study that examined patients’ barriers to telemedicine, which showed that 65% of patients were interested in telemedicine visits but only 54% of patients successfully completed one.(14) Challenges related to internet data access/connection stability (reported in at least 1 in 4 patients), and inability to download video application on the call are common reasons for failure to complete a telemedicine visit.(14) Patient’s age, socioeconomic status, E-health, and computer literacy are common factors that are associated with unsuccessful telemedicine visits.(13) Approximately 25% of Patients who face challenges with connecting to telemedicine visits, often need help from family members for technological support.(14) Notably, the rate of successful completion of telemedicine visits were especially low among non-English and non-Spanish speaking patients’ and patients’ who are > 55 years old. (14), (15),(16) This finding is concordant with our findings, where use of interpreter services for non-English speaking patients was one of the barriers to successful telemedicine visits. Language dis-concordance between physicians and patients is associated with confusion, frustration, and medical errors.(17) Patients with low English language proficiency are known to face significant challenges in navigating the health care system.(17) ,(18),(19) Telemedicine may exacerbate this barrier because patients need to navigate several steps that often require English proficiency (e.g., following instructions to navigate downloading a hospital-approved telemedicine software). Other common issues are delays in connecting with interpreter services during the virtual visit. In addition, some virtual platforms do not consistently have integrated interpreter services available.(17)(19) Thus, use of telemedicine in this population may exacerbate this barrier because patients need to navigate several steps that often require English proficiency (e.g., following instructions to navigate downloading a hospital-approved telemedicine software). (17)

Lastly, patients’ cognitive disability was another challenge that was commonly encountered in telemedicine visits. Although studies examining use of telemedicine in patients with dementia showed promise, adequate support staff and a caregiver that can navigate technology are needed to the success of the telemedicine visit.(20) However, patients with suboptimal support or those with sensory (auditory/visual) or language impairment may not have the same success and subsequently access to health care.(20) Unfortunately, most telemedicine platforms do not have features to facilitate communications with patients who are deaf, blind, or have language or mobility impairment ,or cognitive disabilities.(7), Thus, one must be aware of the possible inequitable access and engagement to health care when telemedicine is used in patients with cognitive disabilities and or physical disabilities.

### Areas of potential improvement to facilitate the use of telemedicine:

Neurologists rated providing adequate administrative support and facilitating ordering ancillary testing (e.g., EEG, EMG, etc.) as the most important issues to be addressed to optimize the use of telemedicine. In our study, nearly half of neurologists reported that their department did not develop a telemedicine protocol or provide virtual workflow training. Examples of common administrative issues that are reported in the literature include missing or incorrect patient’s contact information and lack of patients preparation for the visit.(6) Both, suboptimal virtual work-flow and administrative support can cause negative physicians’ and patients’ experiences, and generate more work for physicians.(5) Therefore, establishing a virtual-workflow and training for staff to support telemedicine visits is important to optimize both physicians’ and patients’ experiences.(5) Lastly, the integration of video and telephone calls into the EMR was also one of the most commonly reported issues to be addressed to improve neurologists’ experiences with telemedicine. One of the organizational challenges to adopting telemedicine is the electronic platform used. During the last three years, there has been an exponential increase in the different software and electronic platforms developed for telemedicine.(21) The decision of which platform to purchase is often met with hesitancy because of the variability in functionality and cost. Video-conferencing function integrated into EMR has the advantage of being more functional and minimizes physicians’ and administrative staff burden to learning different software platforms.

## Practice change implications:

Major opportunities to improve the quality of telemedicine in neurology include developing an efficient EMR system with integrated video-telephone, creating an effective virtual workflow, and ensuring the availability of adequate administrative staff support. To address limitations in physical examination, health systems may adopt a triaging system to determine which patients are likely to need in-person evaluations and to adopt new technological tools to augment the virtual neurologic examination. Finally, to minimize disparities in health delivery, health systems should invest in providing built-in interpreter services and support for patients with difficulty accessing technology or require special assistance due to cognitive disabilities.

Our study limitations include its purely descriptive nature, small sample size that limited the ability to analyze based on subspeciality, inclusion of only neurologists practicing in academic institutions, lack of response rate calculation and the unvalidated survey. In addition, we did not include neurologists’ demographic data (e.g., gender, years of experience), and did not dichotomize inpatient versus outpatient experiences. These limitations may reduce generalizability.

## Figures and Tables

**Table 1:** Participants’ characteristics:

Characteristic:	N (%)

Position at the hospital	94 (85%)
Attending physician	1 (1%)
Advanced practice provider (NP, PA) *	12 (11%)
Fellows	2 (2%)
Residents	1 (1%)
Advanced Nurse practitioner	1 (1%)
Others	

Age group	43 (39%)
30–39	39 (35%)
40–49	15 (14%)
50–59	8 (7%)
60–69	4 (4%)
70 years old or older	

* NP: Nurse practitioner

* PA: Physician assistant

**Table 2:** Patient-related barriers to telemedicine:

Patient-related barriers	N (%)

Technological support to set up telemedicine visit	104 (95%)
Yes	6 (5%)
No	

Patient’s mental/cognitive disability	41 (37%)
Yes	69 (63%)
No	

Interpreter services	41 (37%)
Yes	69 (63%)
No	

Patient’s physical disability	20 (18%)
Yes	90 (82%)
No	

Others (e.g., lack of cooperation, poor internet connection, patients forget their appointments, etc.)	8 (7%)
